# Association of Lipoprotein-associated Phospholipase A2 with Coronary Calcification among American and Japanese Men

**DOI:** 10.2188/jea.17.179

**Published:** 2007-12-19

**Authors:** Aiman El-Saed, Akira Sekikawa, Riad Wahid Zaky, Takashi Kadowaki, Tomoko Takamiya, Tomonori Okamura, Daniel Edmundowicz, Yoshikuni Kita, Lewis H. Kuller, Hirotsugu Ueshima

**Affiliations:** 1 Department of Epidemiology, Graduate School of Public Health. University of Pittsburgh.; 2 Department of Health Science, Shiga University of Medical Science.; 3 Preventive Cardiology, Cardiovascular Institute, University of Pittsburgh Medical Center Health Plan.

**Keywords:** Atherosclerosis, 1-Alkyl-2-acetylglycerophosphocholine Esterase, Coronary Arteriosclerosis, Calcification, Asian Continental Ancestry Group, European Continental Ancestry Group

## Abstract

**BACKGROUND:**

We have previously reported that the prevalence of coronary artery calcification (CAC) was substantially lower among Japanese than American men despite a less favorable profile of many traditional risk factors in Japanese men. To determine whether lipoprotein-associated phospholipase A2 (Lp-PLA2) levels are related to the difference in the prevalence of CAC between the two populations.

**METHODS:**

A total of 200 men aged 40-49 years were examined: 100 residents in Allegheny County, Pennsylvania, United States, and 100 residents in Kusatsu City, Shiga, Japan. Coronary calcium score (CCS) was evaluated by electron-beam tomography, Lp-PLA2 levels, nuclear magnetic resonance (NMR) lipoprotein subclasses, and other factors were assessed in 2001-2002.

**RESULTS:**

Lp-PLA2 levels were higher among American than Japanese men (Mean ± standard deviation 301.7 ± 82.6 versus 275.9 ± 104.7 ng/mL, respectively, p=0.06). Among all Japanese men and those with low density lipoprotein (LDL) cholesterol >130 mg/dL, there was an inverse association of the prevalence of CCS>0 with the fertile groups of Lp-PLA2 levels (p=0.08 and p=0.03, respectively). American men did not have any association between CCS>0 with the fertile groups of Lp-PLA2 (p=0.62). Although Lp-PLA2 among both populations correlated positively with LDL and total cholesterol, American and Japanese men had different correlations with NMR lipoprotein subclasses. Reported high odds ratio for CCS>0 among American compared to Japanese men was not reduced after adjusting for Lp-PLA2 levels.

**CONCLUSION:**

Lp-PLA2 may have different mechanisms of action among American and Japanese men. Lp-PLA2 levels can not explain the observed CAC differences between the two populations.

Lipoprotein-associated phospholipase A2 (Lp-PLA2) is a subtype of the phospholipase A2 superfamily, a family of enzymes that hydrolyze phospholipids, and is secreted by cells of the monocyte–macrophage series, T-lymphocytes, and mast cells. The biological role of Lp-PLA2 has been controversial with seemingly contradictory pro-atherogenic and anti-atherogenic^1^^-^^3^ functions being proposed.

Identifying racial differences in the association of Lp-PLA2 with coronary artery calcification (CAC) or coronary artery disease (CAD) can help to identify populations who can potentially reduce future cardiovascular events by therapeutically lowering baseline Lp-PLA2 levels or by inhibiting Lp-PLA2 activity. Among the Japanese, an inherited deficiency of Lp-PLA2 was reported to be associated with CAD and stroke^4^^-^^7^. However, this mutation has not been found among Caucasians.^8^ Among Caucasians, Lp-PLA2 was reported to be an independent risk factor for CAD and stroke in many,^9^^-^^14^ but not all, studies.^15^^-^^17^ Data assessing the relationship of CAC with Lp-PLA2 are very limited among Caucasians^18^^,^^19^ and there are no data, to our knowledge, among the Japanese.

We have previously reported that the prevalence of CAC was significantly lower among Japanese than white American men aged 40-49 (13% versus 47%, P<0.001), despite a less favorable profile of many traditional risk factors in the Japanese,^20^ which might imply that there are strong protective factors against atherosclerosis among the Japanese. This Japanese paradox is most probably unexplained by genetic differences between the Japanese and Americans as rates of CHD among Japanese Americans living in the United States (US) are much higher than among the Japanese living in Japan.^21^ We examined 200 American and Japanese men. The aim of the study was to compare CAC and risk factors between American men (a high CHD population) and Japanese men (a low CHD population) born after World War II who adopted a western lifestyle. We hypothesized that (1) levels of Lp-PLA2 are much higher among American men, and (2) the higher Lp-PLA2 levels among American men partly explain the higher prevalence of CAC among American men compared to Japanese men.

## METHODS

### Study Population

After obtaining informed consent forms, a total of 200 men aged 40-49 years were examined between 2001 and 2002 as previously described:^20^ 100 American residents in Allegheny County, Pennsylvania, US, and 100 residents in Kusatsu City, Shiga, Japan.

In Allegheny County, subjects voluntarily participated in the study after it was announced with the eligibility criteria through the University of Pittsburgh Medical Center (UPMC) Health Plan. Out of the 100 American subjects, 99 were Caucasians. In Kusatsu City, subjects were randomly selected from the Basic Residents' Register. The participation rate was 49%. Exclusion criteria were (1) clinical cardiovascular disease, (2) type 1 diabetes, (3) cancer except skin cancer in the past two years, (4) renal failure, and (5) genetic familial hyperlipidemias. The study protocol was approved by the Institutional Review Boards (IRB) of the University of Pittsburgh, Pittsburgh, Pennsylvania, US, and Shiga University of Medical Science, Otsu, Japan.

### CAC and Lp-PLA2 Assessment

As previously described,^20^ electron-beam tomography (EBT) scanning was done using a GE-Imatron C150 Electron Beam Tomography scanner (GE Medical Systems, South San Francisco, US) at both sites. Readings of the scanning were done centrally at the Cardiovascular Institute, Pittsburgh, Pennsylvania, US, using the widely accepted Agatston scoring method.^22^ The reproducibility of the EBT scans had an intraclass correlation of 0.99.

Lp-PLA2 levels among both populations were centrally measured using an enzyme-linked immunoabsorbent assay (PLAC test^®^, diaDexus Inc, South San Francisco, California) on fasting plasma samples that were stored frozen at -80°C. The lowest standard limit of detection is 1.3 ng/mL. The inter-assay coefficient of variation was 6.3% and the intra-assay coefficient of variation was 4.3%.

### Assessment of Other Risk Factors

A self-administered questionnaire was used to obtain information on demography, smoking habits, alcohol consumption, and other factors. Body mass index (BMI) was calculated as weight (kg)/height squared (m^2^). Waist circumference was measured at the level of the umbilicus while participant was standing erect. Blood pressure was measured in the right arm of seated participants after the participant emptied his bladder and sat quietly for 5 minutes using a standard mercury sphygmomanometer.

Analysis of frozen samples from the US and Japan were done centrally at US laboratories. Fasting serum lipids, glucose, and insulin were measured at the Heinz Laboratory, Department of Epidemiology, University of Pittsburgh. Serum lipids were measured with standardized methods according to the Centers for Disease Control and Prevention. C-reactive protein (CRP) was measured at the Laboratory for Clinical Biochemistry Research, University of Vermont, by a calorimetric competitive enzyme-linked immunosorbent assay. Lipoprotein subclasses, sizes, and the particle numbers were determined by nuclear magnetic resonance (NMR) spectroscopy at Lipo-Scienec Laboratory, Raleigh, North Carolina.

### Statistical Analysis

One Japanese participant with an almost zero level of Lp-PLA2 was excluded from the analysis due to a possible genetic Lp-PLA2 deficiency. Two Japanese participants without appropriate EBT images and four Japanese participants without Lp-PLA2 data were also excluded from the analysis, resulting in 100 Americans and 93 Japanese. Normally distributed continuous variables were expressed as means ± standard deviation (SD) and were compared using a two-sample t test. Highly skewed continuous variables (CRP and triglycerides) are expressed as median and inter-quartile range and were compared using the Mann-Whitney U test. Categorical data were expressed in percentages and were compared using the Chi-square statistic (or Fisher's exact test when indicated). Correlations of Lp-PLA2 with other continuous variables were calculated using Pearson's (normal data) or Spearman's correlation (skewed data). Correlations of Lp-PLA2 with NMR variables were calculated using Spearman's correlation. All tests were two-tailed and were considered significant if p<0.05.

Because the distribution of coronary calcium score (CCS) was very skewed, the data were analyzed in a categorical form (0 and >0). To compare CAC between American and Japanese men by Lp-PLA2 level, the prevalent CCS>0 was compared among race-specific Lp-PLA2 tertile groups. To test whether Lp-PLA2 levels can explain the observed differences in CAC prevalence between American and Japanese men, odds ratios for CCS>0 among American compared to Japanese men were calculated using logistic regression models after adjustment for age (model 1), age and Lp-PLA2 levels (model 2), age and conventional risk factors (model 3), and age, conventional risk factors, and Lp-PLA2 levels (model 4). SPSS^®^ software (release 14.0, SPSS Inc., Chicago, US) was used for all statistical analyses.

## RESULTS

Lp-PLA2 levels were higher among American men (301.7 ±82.6 ng/mL) than Japanese men (275.9 ±104.7 ng/mL); the difference was marginally significant (p=0.060, Table 1).

**Table 1.** Comparison of major characteristics between the American and Japanese men.^†^

	Americans (n=100)	Japanese (n=93)	p-value
Age (year)	44.6 ±2.9	44.7 ±2.8	0.785
Waist girth (cm)	96.4 ±9.8	84.7 ±8.5	<0.001
Body mass index (kg/m^2^)	27.0 ±3.3	23.3 ±3.1	<0.001
Systolic blood pressure (mmHg)	113.7 ±9.6	122.8 ±14.1	<0.001
Diastolic blood pressure (mmHg)	78.4 ±5.8	78.7 ±10.4	0.785
Total cholesterol (mg/dL)	192.8 ±31.3	221.4 ±37.6	<0.001
Triglycerides (mg/dL) ^†^^†^	116.0 (79.0-167.5)	131.0 (90.0-181.0)	0.172
HDL cholesterol (mg/dL)	45.9 ±11.6	54.8 ±14.9	<0.001
LDL cholesterol (mg/dL)	119.7 ±30.0	136.3 ±39.1	0.001
Fasting glucose (mg/dL)	95.3 ±9.1	103.6 ±8.7	<0.001
Fasting insulin (*μ* IU/mL)	12.5 ±6.6	8.2 ±3.9	<0.001
C-reactive protein (mg/L) ^†^^†^	0.91 (0.45-2.20)	0.43 (0.21-0.86)	<0.001
Coronary calcium score ≥0 (%)	47%	13%	<0.001
Lipid medication (%)	8	3.1	0.134
Current cigarette Smoking (%)	15	48.5	<0.001
Alcohol drinking everyday (%)	16	46.4	<0.001
Lp-PLA2 (ng/mL)	301.7 ±82.6	275.9 ± 104.7	0.06

†: Mean and standard deviation unless mentioned otherwise

††: Median and inter-quartile range

Lp-PLA2: lipoprotein-associated phospholipase A2

Lp-PLA2 levels were negatively correlated to waist circumferences in both American and Japanese men but significant only in American men. Lp-PLA2 levels among both populations had significant positive correlations with both total cholesterol (Americans r=0.37, p<0.001; Japanese r=0.30, p=0.004) and low density lipoprotein (LDL) cholesterol (Americans r=0.44, p<0.001; Japanese r=0.30, p=0.003). However, high density lipoprotein (HDL) cholesterol levels did not correlate with Lp-PLA2 levels among either American (p=0.37) or Japanese men (p=0.50). Total and small very low density lipoprotein (VLDL) cholesterol concentrations positively and significantly correlated with Lp-PLA2 levels among American men. Large LDL concentration and LDL size also positively and significantly correlated while both medium small LDL and medium HDL concentrations negatively and significantly correlated with Lp-PLA2 levels among American men. Only large LDL concentration positively and significantly correlated with Lp-PLA2 levels among Japanese men. Neither CRP nor fibrinogen correlated with Lp-PLA2 levels in either American or Japanese men (Table 2).

**Table 2.** Correlation^†^ of lipoprotein-associated phospholipase A2 (Lp-PLA2) level with coronary risk factor and nuclear magnetic resonance (NMR) lipoprotein subclasses among American and Japanese men.

	Americans (n=100)	Japanese (n=93)
Age (year)	0.02	-0.13
Waist girth (cm)	-0.20	-0.16
Body mass index (kg/m^2^)	-0.15*	-0.12
Systolic blood pressure (mmHg)	0.01	-0.08
Diastolic blood pressure (mmHg)	0.01	-0.12
Total cholesterol (mg/dL)	0.37***	0.30**
Triglycerides (mg/dL) *^b^*	0.06	-0.07
HDL cholesterol (mg/dL)	-0.09	0.07
LDL cholesterol (mg/dL)	0.44***	0.30**
Fasting glucose (mg/dL)	0.02	-0.20*
Fasting insulin (*μ* IU/mL)	-0.11	-0.09
C-reactive protein (mg/L) *^b^*	0.11	0.04
Total VLDL particles (nmol/L)	0.27**	0.02
Large VLDL/chylomicrons (nmol/L)	0.01	-0.07
Medium VLDL (nmol/L)	0.16	-0.07
Small VLDL (nmol/L)	0.32**	0.17
Total LDL particles (nmol/L)	0.14	0.08
IDL (nmol/L)	-0.07	-0.01
Large LDL (nmol/L)	0.52**	0.22*
Small LDL (total) (nmol/L)	-0.17	-0.05
Medium small LDL (nmol/L)	-0.21*	-0.06
Very small LDL (nmol/L)	-0.16	-0.05
Total HDL particles (nmol/L)	-0.10	-0.01
Large HDL (nmol/L)	0.11	0.11
Medium HDL (nmol/L)	-0.25*	-0.12
Small HDL (nmol/L)	-0.10	-0.06
VLDL size (nm)	-0.12	-0.15
LDL size (nm)	0.35**	0.10
HDL size (nm)	0.12	0.18

†: Pearson's rho with normal data or Spearman's rho with skewed data.

*: <0.05, **: <0.01, ***: <0.001

LDL: low density lipoprotein

HDL: high density lipoprotein

IDL: intermediate density lipoprotein

VLDL: very low density lipoprotein

**Figure 1.**
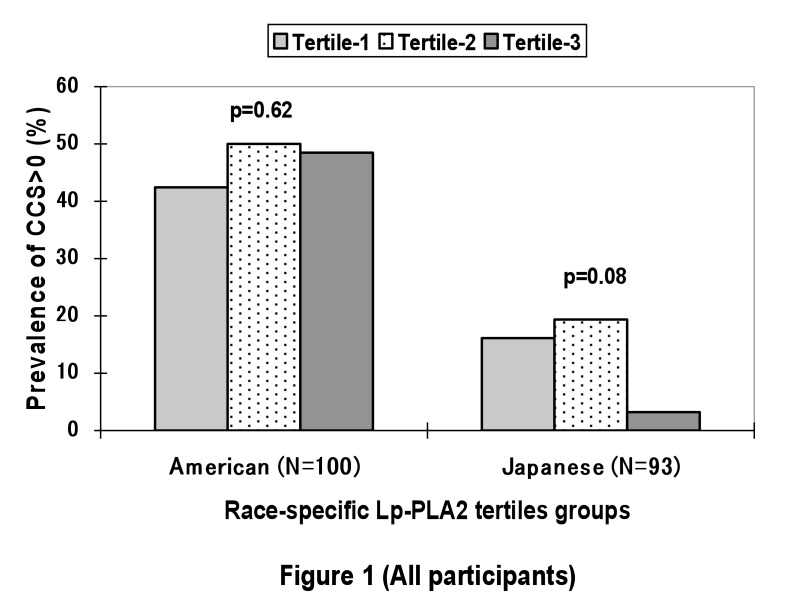
Prevalence of those with coronary calcium score (CCS) > 0 among the 2 populations by tertile group of lipoprotein-associated phspholipase A2 (Lp-PLA2): all participants.

**Figure 2.**
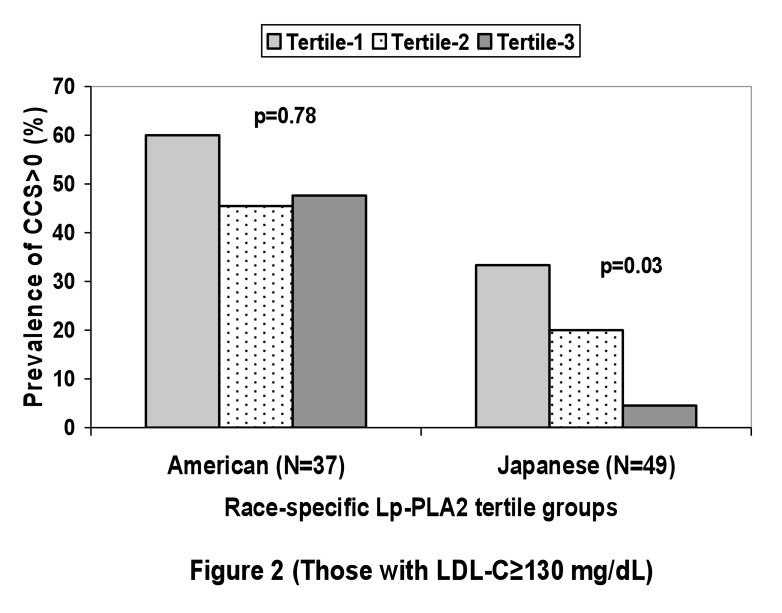
Prevalence of those with coronary calcium score (CCS) > 0 among the 2 populations by tertile group of lipoprotein-associated phspholipase A2 (Lp-PLA2): those with low density lipoprotein cholesterol ≥ 130 mg/dL.

Japanese men had a trend of inverse association of the prevalence of CCS>0 with the tertile groups of Lp-PLA2 levels (p=0.08); the association was significant among Japanese men with LDL?130 mg/dL (N=49, p=0.03). American men did not have any significant associations of the prevalence of CCS>0 with the tertile groups of Lp-PLA2 (p=0.62) (Figures 1 and 2).

Age-adjusted odds ratios for CCS>0 among American compared to Japanese men were highly significant before (6.0, 95% CI 2.9, 12.2, p<0.001) and after (6.4, 95% CI 3.0, 13.3, p<0.001) adjusting for Lp-PLA2 levels. Similarly, odds ratios for CCS>0 among American compared to Japanese men adjusted for multiple risk factors (age, systolic blood pressure, blood glucose, smoking, alcohol, BMI, waist circumference, CRP, total, LDL and HDL, triglycerides, and lipid medication) were highly significant before (7.6 95% CI 2.4-23.8%, p=0.001) and after (8.7 95% CI 2.5-29.7%, p=0.001) adjusting for Lp-PLA2 levels.

## DISCUSSION

The current data showed marginally significantly higher levels of Lp-PLA2 among American compared to Japanese men. There was no significant association between Lp-PLA2 levels with the prevalence of CAC among American men while there appeared to be a negative association between Lp-PLA2 levels with the prevalence of CAC among Japanese men, especially among those with LDL > 130 mg/dL.

With few exceptions,^15^^-^^17^ the majority of clinical and epidemiologic studies among western populations suggested that Lp-PLA2 is an independent predictor of cardiovascular events (CAD and stroke).^9^^-^^14^ Whether Lp-PLA2 is an independent predictor of CAC or not is less clear. Only two studies examined the association between coronary calcification and Lp-PLA2 levels among white populations with inconsistent findings.^18^^,^^19^ In a recent report from the Rotterdam Study,^19^ Lp-PLA2 activity measured concurrently to EBT scanning was not associated with coronary calcification in either men or women. This finding was in contrast to previous findings from the same population that showed Lp-PLA2 as a predictor of CHD and stroke.^10^ In the Coronary Artery Risk Development in Young Adults (CARDIA) study,^18^ however, Lp-PLA2 level had a significant and independent association with CAC among young white and black American men and women.

The negative association between Lp-PLA2 levels and the prevalence of CAC among Japanese men supports the suggested anti-atherogenic effect of Lp-PLA2 among Japanese.^23^ The anti-atherogenic properties of Lp-PLA2 are thought to be due to the degradation of the potent pro-inflammatory phospholipid platelet-activating factor (PAF), possibly through its partial association with HDL cholesterol, as well as the enzymatic catabolism of biologically active oxidized phospholipids in LDL cholesterol.^1^^-^^3^ Moreover, deficiency of enzymatic activity resulting from single nucleotide polymorphism in the Lp-PLA2 gene was reported to be associated with increased risk of developing atherosclerosis and its clinical manifestations.^4^^-^^7^

In this report, the risk of having CAC was several times higher in American men compared to Japanese men before and after adjusting for Lp-PLA2 levels. The findings suggest that the higher Lp-LPA2 levels among American compared to Japanese men cannot explain the higher prevalence of CAC among American men. In this study, among both populations, neither CRP nor fibrinogen were correlated with Lp-PLA2 levels, which is in accordance with previous studies.^9^^,^^10^^,^^16^^,^^18^

One major strength of this study is the ability to examine the association between Lp-PLA2 levels and NMR lipoprotein subclasses, which could help with understanding the Lp-PLA2 mechanism of action. This is because the majority of circulating Lp-PLA2 in plasma is associated with LDL particles.^11^ Although Lp-PLA2 among both populations in this report correlated positively with LDL and total cholesterol as measured by traditional chemical methods, lipoprotein particle sizes and concentrations as measured by NMR spectroscopy correlated differently with Lp-PLA2 levels among Japanese and American men, suggesting that Lp-PLA2 could have different mechanisms of action. Other strengths include comparing two populations with different cardiovascular risk,^20^ the lack of interference of treatments (only 5.7% of all participants were using cholesterol lowering agents), and the availability of multiple established cardiovascular risk factors. There are several limitations in this study: (1) the small sample size of this study; however, the data may serve as a pilot study for future large scale prospective studies comparing Japanese-white differences in the relation between Lp-PLA2 and CAC or CAD ; (2) the observational and cross-sectional nature of the data, precluding causal inferences; and (3) the fact that American subjects were volunteers and not a randomly selected population.

In summary, Lp-PLA2 levels cannot explain the observed CAC differences between the two populations. Lp-PLA2 was not associated with CAC among American men and appeared to be negatively associated with CAC among Japanese men. American and Japanese men had different correlations with lipoprotein subclasses, suggesting different Lp-PLA2 mechanisms of action among the two populations. These data need to be confirmed by large scale prospective studies before any clinical implications are anticipated.
